# Fidelity evaluation of the compared procedures for conducting the PVS-PREDIAPS implementation strategy to optimize diabetes prevention in primary care

**DOI:** 10.1186/s12875-021-01378-z

**Published:** 2021-02-11

**Authors:** Alvaro Sánchez, Heather L. Rogers, Susana Pablo, Esther García, Inmaculada Rodríguez, Mª. Antonia Flores, Olga Galarza, Ana B. Gaztañaga, Pedro A. Martínez, Eider Alberdi, Elena Resines, Ana I. Llarena, Gonzalo Grandes, on behalf of the PREDIAPS Group, Amaia Bengoetxea, Olga Galarza, Elsa Martínez, Itziar Zalduegi, Dorothea Chausson, Agurtzane Gorroño, Alicia Pollán, Marisol Bernabéu, Mª Yolanda Calvo, Ander Artiagoitia, Nerea Zaramillo, Lidia Gonzalez, Asier Aurrekoetexea, Jon Azkarate, María Muñoa, Mar Bilbao, Vicki Camineiro, Gonzalo Gómez de Iturriaga, Fernando Gago, Iciar Ochoa de Retana, Ana Zorrilla, Mª Luisa Gutiérrez, Jone Capetillo Serra, Mª. Nieves Lopez, Nekane Iguerregui, Dolores López, Maite Gastañaga, Antonia Flores, Marcos Pereda, Amaya González, Ana Castresana, Laura Saiz, Nerea Regulez, Estibaliz Peciña, Jasone De la Plaza, Lucía Irastoza, Jose Contreras, Idoia Etxebarria, Begoña Oleaga, Cristina Herrero, Nora Cabezón, Fátima Calvo, J. Manuel Llamazares, Mª. Ángeles Gutierrez, Monica Prieto, Concepción Estébanez, María José Cordovilla, Alicia Domínguez, Isabel Lázaro, Elena Resines, Yolanda Villalba, Begoña Ayerdi, Florencia Martín, Magdalena Presmanes, Floreal Crespo, Araceli Benito, Mª. Belén Molina, Mª. Mar García, Mª. Gracia Díaz, Mª. Luisa Rodriguez Ortiz de Zarate, Rebeca San Cristóbal, M. Zugazaga Prieto, Pedro Martínez, Mercedes Crespo, Estíbaliz Albitre, Adelina García-Roldán, Amaia García, Maite Castro, Iñaki Gorospe, Amelia V. Hernández, Maite López, Mirian Sainz, Irene Marín, María Jesús Aragón, Leire Ulayar, Encarnación Santamaría, Carmen Sánchez, Javier Bayo, Begoña Urkullu, Ana Inés Pereda, Mercedes Garcia, Pilar Blanco, Silvia Soler Valverde, Jose I. Atela, Hiart Trespalacios, Anabel Llarena, Verónica Ruiz, Begoña Cabieces, Concepción Ugarte, Guadalupe Icaza, Edurne Zubeldia, Idoia González, Ángeles Gayo, Itxaso Arévalo, Gloria Intxausti, Esther García, Teresa Sánchez, Igone Lobato, Noelia Fuente, Naiara Ortolachipi, Edelweiss Sánchez, Victoria Cosgaya, Ángeles Gayo, Arantza Azazeta, Patricia Zaballa, Ana Isabel Ramila, Teresa Rodeño, Inmaculada Rodríguez, Teresa Vázquez, Raquel Ruíz, Rosa Herrero, María Valvanera, Saioa Setién, Begoña Ruíz, Juan José Casas, Joana Clemente, Javier Amiama, Javier Angulo, Belén Aramendia, Soledad Asenjo, Mª. Sonia Mayoral, Remedios Oyarzun, Bergoi Calvar, Belinda Zulueta, Edurne Elola, Josu Egaña, Gemma Díaz, Mª. Ángeles Sola, Laura Balague, Mª. Ángeles Ganzarain, Arantxa Aramburu, Ana Mª. Guinea, Edurne Lizarazu, Inma Valverde, Nekane Arenas, Susana Alonso, Rosa Salaberria, Javier Merino, Mercedes Álvarez, Ester Lázaro, Juncal Izcara, Leire González, Ainhoa García Leunda, Idoia Sánchez, Esther Usandiaga, Eluska Yetano, Jaione Larrea, Inés Mendinueta, Asún Uria, Ana Belén Gaztañaga, Eva Mayo, Onintza Aranzadi, Eulalia Medina, Rosa González, Ione Gutiérrez, Arantxa Perez, Mª. Ángeles Izquierdo, Alejandro García, Ainhoa Ugarte, Mª. Teresa Zubeldia, Bingen Uriondo, Mª. Carmen Aranegui, Arantxa Mendiguren, Yolanda Fernández, Maite Zapirain, Mª. Jose Garín, Aitziber Ayerbe, Jon Urkia, Alvaro Sánchez, Josep Cortada, Susana Pablo, Heather Lynn Rogers, Arturo García-Alvarez, Gonzalo Grandes, Alicia Cortazar, Patxi Ezkurra, Rafa Rotaetxe

**Affiliations:** 1 Primary Care Research Unit, Deputy Directorate of Healthcare Assistance, Biocruces Bizkaia Health Research Institute, Basque Healthcare Service – Osakidetza, Plaza Cruces s/n, E-48903 Barakaldo, Spain; 2 Biocruces Bizkaia Health Research Institute and Ikerbasque Basque Foundation for Science, Bilbao, Spain, Plaza Cruces s/n, E-48903 Barakaldo, Spain; 3 Zalla Primary Care Center, Plaza Euskadi s/n, E-48860 Zalla, Spain; 4 Sodupe Primary Care Center, Sodupe Goikoa 13, E-48830 Gueñes, Spain; 5 Erandio Primary Care Center, Ibarrondo s/n, E-48950 Erandio, Spain; 6 Alango Primary Care Center, Alango 30, E-48992 Getxo, Spain; 7 Egia Primary Care Center, Ametzagaina 8, E-20012 Donostia-San Sebastian, Gipuzkoa Spain; 8 San Vicente Primary Care Center, Elejalde s/n, E-48902 Barakaldo, Spain; 9 Iztieta Primary Care Center, Avda. Lezo s/n, Errenteria, E-20100 Gipuzkoa, Spain; 10 Zuazo Primary Care Center, Lurkizaga s/n, E-48902 Barakaldo, Spain; 11 Portugalete Primary Care Center, General Castaños 34, E-48920 Portugalete, Spain

**Keywords:** Fidelity, Implementation strategy, Diabetes prevention, Primary healthcare

## Abstract

**Background:**

Assessing the fidelity of an implementation strategy is important to understand why and how the strategy influences the uptake of evidence-based interventions. The present study aims to assess the fidelity of the two procedures for engaging primary care (PC) professionals and for the deployment of an implementation strategy for optimizing type 2 diabetes prevention in routine PC within the PREDIAPS randomized cluster implementation trial.

**Method:**

We conducted a mixed-method fidelity evaluation study of the PVS-PREDIAPS implementation strategy. Nine PC centers from the Basque Health Service (Osakidetza) were allocated to two different procedures to engage physicians and nurses and deploy a implementation strategy to model and adapt the clinical intervention and its implementation to their specific contexts: a Global procedure, promoting the cooperation of all healthcare professionals from the beginning; or a Sequential procedure, centered first on nurses who then pursued the pragmatic cooperation of physicians. Process indicators of the delivery and receipt of implementation strategy actions, documented modifications to the planned implementation strategy, and a structured group interview with centers’ leaders were all used to assess the following components of fidelity: adherence, dose, quality of delivery, professionals’ responsiveness and program differentiation.

**Results:**

Generally, the procedures compared for professionals engagement and deployment of the implementation strategy were carried out with the planned differentiation. Nonetheless, some unexpected between-group differences were observed, the initial rate of collaboration of nurses being higher in the Sequential (93%) than in the Global (67%) groups. Exposure rate to the programed implementation actions (% of hours received out of those delivered) were similar in both groups by professional category, with nurses (86%) having a higher rate of exposure than physicians (75%). Professionals identified half of the planned discrete strategies and their rating of strategies’ perceived usefulness was overwhelmingly positive, with few differences between Sequential and Global centers.

**Conclusions:**

The PVS-PREDIAPS implementation strategy has been implemented with high fidelity and minor unplanned reactive modifications. Professionals’ exposure to the implementation strategy was high in both groups. The centers’ organizational context (i.e., work overload) led to small mismatches between groups in participation and exposure of professionals to implementation actions.

**Trial registration:**

Clinicaltrials.gov identifier: NCT03254979. Registered 16 August 2017.

## Background

Implementation fidelity has, until recently, been defined as the degree to which a given program is implemented as it was originally planned and it has been mostly focused in measuring the deployment of the evidence-based intervention under study [1, 2]. Yet, in complex interventions, the program includes the evidence-based intervention and the supporting implementation strategies aimed at facilitating the adoption of this intervention by those responsible for delivering it [3, 4]. Consequently, factors related to adherence to the planned implementation strategy, dose received, i.e., the extent to which the recipient was exposed to the implementation strategy, participant responsiveness and actual involvement, as well as modifications made and the role of context, become central issues for understanding the impact of implementation initiatives to improve real-world clinical practice [3, 5]. As the field of implementation expands and the use of hybrid trial designs grows [6], the distinction between intervention-level fidelity and implementation strategy-level fidelity is becoming increasingly important.

Evaluating the degree to which implementation strategies are operated as designed within implementation trials are key in order to determine the internal and external validity of implementation studies [1, 3–5]. They are necessary to investigate both the receipt and the scope of an implementation strategy to improve the adoption of evidence-based practice in routine settings [4, 7]. Additionally, they help in the interpretation of outcome results of interventions translated to real practice and inform the optimization of both the clinical intervention and/or implementation strategy to favor adoption of the intervention and implementation and future scale-up in other contexts and settings [8–11]. Fidelity evaluation is especially necessary in multisite trials, where the “same” implementation strategy may be enacted and received in different ways [12, 13].

However, despite the importance of implementation fidelity evaluation, first, there is currently no framework explicitly establishing either a set of procedures or specific requirements to guide the evaluation of the fidelity of an implementation strategy [4, 14]. And second, likely linked to this first issue, under-reporting of fidelity of implementation strategies is the rule rather than the exception in the implementation research literature [4]. Among general existing frameworks to guide fidelity evaluations [2, 3, 5, 7, 8], the framework stated by Dane et al. [8] and its adaptation by Dusenbury et al. [5], has been successfully used by others for rating the fidelity of implementation strategies [4]. This framework points to the following five main fidelity dimensions: a) “adherence”, which strategies were actually used during implementation versus which were planned; b) “dose”, the quantity of the implementation strategy delivered and extent to which the recipients actually received it; c) “quality of program delivery”, how well the components of the implementation strategy have been delivered and if modifications occurred; d) “participant responsiveness”, how the delivery process of an implementation strategy is received by its recipients; and e) “program differentiation”, defined as the degree of enactment of differentiated procedures or strategies in one condition from that in the other condition should be also considered as an important element of implementation fidelity evaluation [4, 5, 8].

The aim of the present study is to evaluate the fidelity of two procedures being compared for deploying the PVS-PREDIAPS implementation strategy to optimize type-2 diabetes prevention in primary care (PC) [15]. Briefly, the PVS-PREDIAPS implementation strategy consist in conducting externally facilitated collaborative modeling process through a set of planned implementation actions with PHC professionals in order to adapt and integrate an evidence-based healthy lifestyle promotion intervention to prevent type-2 diabetes within the routine primary care services. This fidelity evaluation will help to gain understanding about the quality of the implementation of the PVS-PREDIAPS strategy and specifically regarding the two procedures compared for engaging professionals and deploying the implementation actions. This in turn will help to explain and interpret future results of the PREDIAPS trial (ie., to reject a possible type III implementation failure error), will identify what has been changed from the original implementation plan and how changes may impact outcomes, and will inform how future dissemination and scale-up can be improved. Specifically, the objectives of the present study were to:

- To describe the process indicators of the PREDIAPS trial and deployment procedures of the PVS-PREDIAPS implementation strategy [15], including a clear description of the context in which it has been implemented
- To assess the fidelity of the delivery of the PREDIAPS implementation strategy among the groups compared, in terms of the dose of both the strategy delivered and that actually received by professionals and centers involved, the planned and unplanned modifications that took place, i.e., adherence, and the degree to which elements can reliably differentiate one type of procedure for engagement and deployment of the strategy from another, i.e., program differentiation.
- To assess the perceived usefulness of the implementation strategy by professionals involved (responsiveness)”

## Methods

### Design

This is a mixed-method fidelity evaluation study of the 2.5-year enactment of an implementation strategy to improve the adoption of an evidence-based healthy lifestyles intervention for the prevention of type-2 diabetes in primary care - the PREDIAPS Trial. Briefly, the PREDIAPS is a randomized cluster implementation trial conducted in nine Basque Health Service (Osakidetza) PC centers that aims to assess the effectiveness and feasibility of different engagement procedures to perform a facilitated interprofessional collaborative process - the PVS-PREDIAPS implementation strategy- to optimize type-2 diabetes prevention in routine PC. The protocol has been published elsewhere [15].

Headed by a local leader and an external facilitator, centers were expected to perform a collaborative structured process to adapt an evidence-based intervention to promote healthy lifestyles and its implementation to their specific context. Centers were randomly allocated to one of two arms. One arm was to apply this strategy to engage staff globally, promoting the cooperation of all healthcare professionals from the beginning, while the other arm applied the strategy sequentially, centering first on nurses, who then sought the pragmatic cooperation of physicians. The research protocol was approved by the Basque Country Clinical Research Ethics Committee (Ref. no.: 08/2015) and the protocol was registered in Clinicaltrials.gov (identifier: NCT03254979, registered 16 August 2017).

### Participants

PC centres: Osakidetza PC centers identified by managers as having adequate organizational readiness for change seeking to optimize the primary prevention of T2D were considered eligible. Centers were finally included if, after a session introducing the intervention, they succeeded in gaining written consent and agreement to participate from the majority (> 51%) of the nursing staff of the center and a substantial proportion of the physicians whose patients would be involved through the nurses.

PC Users: Patients aged 30 years or more who sought medical attention at least once through the participating centers between 2 March 2017 and 2 March 2018, and had been classified as at high risk of developing T2D and/or prediabetes (abnormal fasting glucose level or glucose intolerance plus an additional known cardiovascular risk factor) but did not have a documented diagnosis of T2D in their health record were eligible to participate in the T2D prevention program at the centers. The final definition of the target population was the result of actions implicit in the implementation strategy.

### Recommended evidence- and clinical practice guideline-based clinical intervention

A program was recommended based on scientific evidence and the clinical practice guidelines available, reviewed by our research group in 2016 [16]. This program involved first screening for T2D risk. Then, patients identified as at high risk should be invited, and if they agree, participate in an intensive structured intervention program focused on the prescribing of personalized plans for lifestyle change (low-energy low-fat diet or Mediterranean-type healthy diet; and at least 150 min of moderate physical activity a week). Lastly, patients should be followed up, initially with frequent contact and then annual check-ups. The 5 A’s (Ask, Advise, Agree, Assist, and Arrange follow-up) intervention framework was used to standardize the provision of the evidence-based behavior modification techniques used to promote changes in physical activity (150 min of moderate physical activity a week) and diet (Mediterranean-type healthy diet) to prevent type-2 diabetes in high-risk patients (see Appendix Table 4).

### Implementation strategy for facilitating the adoption of the recommended intervention in routine clinical practice

The PVS-PREDIAPS strategy is based on the creation of an inter-professional community of practice that undertakes a process of modelling, adapting and integrating the recommended clinical intervention into the local context, led by the clinicians themselves, a local leader and an external facilitator [15]. This translates into the following actions to be carried out over the course of an implementation phase lasting 1 year (including holiday periods):

- Selection by consensus in the center and subsequent **training of the local leader** (3 sessions, 15 h in total). The goal is for the leader to acquire knowledge and skills concerning the primary prevention of T2D and planning for the implementation of primary prevention in practice, as well as communication and leadership skill and techniques. Further, seeking to offer ongoing support and facilitation to the local leaders, 4-h **Ongoing leader support meetings** are to be held monthly, for preparing the actions to be taken in each of the sessions held in the centers and reviewing the progress made.
- **Training for the clinical intervention** (90-min theoretical and practical training session) and the software support tool (6-h practical training session on the PVS-OSABIDE_AP tool)
- **Collaborative modeling of the local T2D prevention program**: creation of an inter-professional community of practice lead by the local leader and external facilitator, completion of a needs assessment and process to identify areas for improvement, and mapping of actions, parties responsible, flows and procedures for the intervention program (three 90-min study, discussion and consensus sessions)
- **Plan-Do-Study-Act (PDSA) piloting cycles**: process involving short pilot studies of specific actions seeking to assess their efficiency and, thereby, optimize the set of actions in the program (each cycle involving a 90-min session for planning and/or evaluation of the cycle)
- **Standardization and integration of the program**: final specification of the objectives, intervention target population and screening or identification strategy, key components of the intervention and follow-up: detailed mapping of actions and processes; staff involved, material and organizational components, as well as components related to the context of the center, etc. (one 90-min study, discussion and consensus session)

The full set of actions of the implementation strategy take a total of 20 h. Once the program has been deployed in the centers and seeking to assess progress and provide ongoing support to encourage intervention programs being sustainably integrated into each center’s portfolio of services, a set of **Regular audits and ongoing facilitation sessions** are to be held over the second year post-implementation (six 90-min sessions, a session every 2 months). The specific discrete implementation strategies used in the PREDIAPS trial, as cataloged by the Expert Recommendations for Implementing Change (ERIC) taxonomy [17], are described elsewhere [15].

### Random allocation to the procedures compared

As described elsewhere [15], with the aim of isolating the effect of two different procedures to engage professionals and perform the PVS-PREDIAPS facilitated interprofessional collaborative implementation strategy to optimize T2D prevention in routine PC, centers were randomly assigned to: a Global strategy, seeking involvement and cooperation between physicians and nurses from the outset; or a Sequential strategy, first led by nurses, and then seeking the pragmatic involvement and cooperation of physicians later in the process. Specifically, physicians within the Sequential group were involved from the second PDSA cycle, and consequently, they only participated in 4.5 h of the core implementation strategy actions estimated to take 20 h in total. The allocation process was conducted using a random number sequence generated by computer prior to the start of the trial by an external researcher from the Primary Care Research Unit of Bizkaia.

### Measures

In order to evaluate the fidelity of the two procedures to perform the PVS-PREDIAPS collaborative modeling implementation strategy to optimize T2D prevention in PC [15], the following factors were measured in accordance to the fidelity evaluation framework stated by Dane [8] and Dusenbury [5].

#### Adherence

The degree of adherence to the planned execution of the implementation strategy for each procedure for conducting the PVS-PREDIAPS implementation strategy and its active ingredients are assessed by comparing three sources of information: i) the protocol for the implementation strategy [15]; ii) actual process indicators; and, iii) the reports, material and products resulting from the sessions. The FRAME framework [18] for reporting modifications (planned or reactive) to evidence-based interventions is used in order to evaluate and describe modifications made to the planned implementation strategy. Specifically, for each of the modifications, the following were specified: the reason, by whom they was requested or encouraged, when and how they took place, and the degree to which they affect fidelity depending on whether they were (or were not) planned in advance. In addition, a strategy mapping for complex interventions [19] has been used to specify the strategies deployed and the timing in which the strategies have been applied.

#### Dose

The following indicators related to the process of conducting the PREDIAPS trial and the actions embedded to deploy the implementation strategy are specified for reporting on the dose:

- Percentage of centers included out of all those approached
- Percentage of healthcare professionals who initially collaborated out of the total number of professionals at each center
- Actions carried out over time (training, work sessions, etc.).
- Participation of collaborating healthcare professionals in each action and actual exposure to the implementation strategy actions compared to that originally planned (% of hours/action received out of the total number of hours that would be implied by participation in all the actions of the strategy, i.e., 20 h or 4.5 h).

#### Quality of program delivery/participant responsiveness

A structured group interview was carried out with the local leaders from the centers involved (*n* = 9) in order to assess the perceived usefulness of the implementation strategy among healthcare professionals. Six open-ended questions focused on the implementation strategies perceived to be part of the PREDIAPS trial, the perceived value of these strategies, and recommendations for their optimization. This interview was video-recorded with prior consent from the participants. Specifically, the following questions were used:

a) personal rating of the implementation process and associated strategies (through questions such as, “*Specifically, which aspects of the process for optimizing practice do you consider the most important or useful for doing your job?*”)

b) recommendations for optimizing the PVS-PREDIAPS implementation strategies for achieving the following among the staff: (i) build personal competence, (ii) engage staff and (iii) enhance interprofessional cooperation (through questions such as, “*In your opinion, which aspects should be strengthened to build competence among staff for putting the innovation into practice in a sustainable way in your center.*

#### Program differentiation

Lastly, program differentiation is determined by comparing the degree to which indicators of adherence, dose and modifications can be reliably differentiated between one type of procedure for engagement and deployment of the strategy and another [5].

#### Other measured variables

The following variables were used in order to describe collaborating centers’ characteristics: (1) center size, measured in terms of the number of users assigned to receive care at each center (the catchment population) and the number or practitioner lists; (2) center location (Suburban, City center or Town-rural); and (3) the aggregated socioeconomic status of people assigned to each center as measured by the Deprivation Index [20]. This index is an ordinal variable, categorized into five levels (deprivation quintiles; 1 representing high and 5 low socio-economic status), providing a relative measure of the socioeconomic characteristics of the population of census tracts. Its design allows socioeconomic and environmental inequalities between residents to be estimated by census tract in Spain. The calculation takes into account the percentages of residents in a tract who, according to the most recent data available (2016 census), are manual workers, unemployed, or on temporary contracts, or overall or specifically among young people, have a low level of educational attainment.

### Analysis

Frequencies and proportions were used to describe characteristics and process indicators related to professional participation and exposure rates for each collaborating center and for the Global and Sequential groups. The mean deprivation quintile for all patients under the care of each of the collaborating centers in 2016 was used to estimate the Deprivation Index at center level.. Regarding the qualitative study based on a structured group interview, responses to the questions were extracted from both the paper surveys and the audio of the discussion that emerged. For the analysis and interpretation of this information, the ERIC strategies [17] referred to in the healthcare professionals’ responses were identified and coded positively or negatively. These strategies were then classified by center and question grouping.

## Results

Of the 12 PC centers put forward by the management of the 4 participating Osakidetza integrated healthcare organizations, 9 were recruited to the project. Of these nine centers, six were classified as being located in an “Urban city” area and three were allocated to each of the comparison groups, while the only center classified as “Residential”, which had the lowest mean Deprivation Index, was allocated to the Global strategy group, and the only two “Urban-rural” centers were allocated to the Sequential strategy group. Centers described as “large” based on the catchment population and number of practitioner lists were more represented in the Global than in the Sequential group, which contained the two smallest centers.

Across the 9 centers, 137 physicians and nurses originally gave written consent and agreed to collaborate (70% of all the physicians and 82% of all the nurses assigned to these centers) (Table 1). The initial collaboration rate among nurses was higher in the Sequential (94%) than in the Global (69%) group. The overall exposure rate to the programed implementation strategy actions (% of hours received out of those delivered; maximum = 20 h) was slightly higher in the Sequential than the Global group for both categories, with nurses (89.4% vs 85%) having a higher rate of exposure than physicians (82.6% vs 75%). Assessing the exposure of collaborating professionals in each category to the implementation actions, the percentage of nurses exposed to at least 80% of the actions was again a higher in the Sequential group (70% vs 60% in the Global group).

**Table 1:** Collaborating primary care centers’ characteristics and professionals participation and exposure rates

	Global Group					Sequential Group					
	Center 1 A^†^	Center 2 Zu	Center 3 I	Center 4 P	*TOTAL*	Center 5 Er	Center 6 Sv	Center 7 Eg	Center 8 Za	Center 9 So	*TOTAL*
Type of PC	Sub-urban	City center	City center	City center	*NA*	City center	City center	City center	Town-rural	Town-rural	*NA*
Deprivation Index of the population, mean	1.5	3.9	3.5	2.9	*NA*	3.9	3.8	2.8	2.8	2.9	*NA*
Catchment population, n	15,201	16,552	22,438	14,897	*69,088*	12,348	17,877	12,948	10,324	7374	*60,871*
Initial collaboration from the centers’											
Physicians, n/total	10/12	13/14	8/15	6/11	*37/52*	6/7	7/9	6/9	4/5	5/5	*28/35*
Nurses, n/total	8/12	8/13	14/15	5/11	*35/51*	7/7	9/9	9/9	4/6	5/5	*34/36*
Practitioner lists, n	10	12	7	6	*35*	8	8	9	6	5	*36*
Exposure rate*											
Physicians, %	72%	66%	78%	84%	*75%*	85%	82%	80%	77%	89%	*82.6%*
Nurses, %	90%	89%	79%	82%	*85%*	90%	81%	86%	98%	92%	*89.4%*
% professionals exposed to 80% of the strategy											
Physicians, %	30%	15%	37%	83%	*41.2%*	33%	37%	14%	25%	80%	*37.8%*
Nurses, %	62%	75%	43%	60%	*60%*	86%	55%	55%	75%	80%	*70.2%*

^†^ Primary Care centers’ name initials: *A* Alango, *Zu* Zuazo, *I* Iztieta-Errenteria, *P* Portugalete, *Er* Erandio, Sv San Vicente, *Eg* Egia, Za Zalla, So Sodupe

* Exposure rate in physicians is calculated as the percentage of hours pertaining to implementation actions received by the professionals from the total number of action/hours planned, being the total hours 20 h for physicians in the global group and nurses of both group, and 4.5 h for physicians in the Sequential group; Considering all professionals that have been involved as staff turnover has occurred randomly in the course of the process

Tables 2 and 3 describes the actual execution of the actions of the implementation strategy and its components, including modifications (planned or reactive), over the course of the 2.5 years during which the deployment of the strategy and setting up of the intervention program were due to take place (March 2017–May 2019). All nine centers held the three leader training sessions and the 10 sessions led by the local leader supported by the external facilitator, which comprise the core strategies and actions within the implementation phase. The rates of participation in each of the sessions of the strategy were over 50% of the staff in each category in almost all cases and confirm differing patterns of execution of the actions by centers and by group (Global vs Sequential) (see Appendix Fig. 1). In the post-implementation phase, five sessions were held to support and monitor the setting up of the intervention program. As a component of the strategy, befovre each of the sessions held in their center,11 coordination and preparation sessions were run for the leaders, led by the facilitator, of which 5 were for following-up the setting up of the program and monitoring progress.

**Table 2:** PVS-PREDIAPS implementation strategy and action timing mapping

YEAR 1 (March 2017–March 2018)		MONTH											
**Implementation strategy component**	**Actions conducted**	1 Mar	2 Apr	3 May	4 June	5 July	6 Aug	7 Sept	9 Oct	9 Nov	10 Dec	11 Jan	12 Feb
**Strengthening of Local Leadership** *Goals****:*** *to provide the local coordinator with interpersonal and organizational skills to support the implementation Discrete implementation strategies: Recruit, designate and train for leadership (ERIC 57); Conduct educational meetings (ERIC 15); Ongoing support for implementation (ERIC 55)*	***3-day leader training workshop*** (Lt _i_) (5 h/session)	X _Lt1-3_											
	**Ongoing leader support meetings** (Lsm_i)_ (4 h/meeting)		X_Lsm1_	X_Lsm2_				X_Lsm3_		X_Lsm4_	X_Lsm5_		
**Training in the clinical intervention** *Goals: to provide initial training in the recommended type 2 diabetes (T2D) prevention clinical intervention and the ICT support tool in the electronic health record Specific implementation strategies: Conduct educational and skill development meetings (ERIC 15 and 19); Changes in record systems (ERIC 12)*	***Session 1****: Primary prevention of T2D in PC: evidence and recommended practice (90 min/session)*	X_s1_ *M*_*1*_ X_s2_											
	***Session 2****: ICT application for the promotion of healthy habits in the EHR (6 h/session)*												
**Collaborative planning of the intervention program** *Goals: to plan the local program based on shared decision-making: objectives, actions, agents, work flow, organization and sharing out of tasks Discrete implementation strategies: Conduct local needs assessment (ERIC 18); Conduct educational and outreach meetings (ERIC 15); Conduct local consensus discussions (ERIC 17); Intervention mapping (ERIC 48, 51, and 59); Conduct ongoing training (ERIC 19); Plan-Do-Study-Act cycles (ERIC 14); Develop a formal implementation blueprint (ERIC 23).*	***Session 3*** *– Needs assessment and prioritization of areas for improvement (90 min);*			X_s3_									
	***Session 4/5*** *– Planning T2D prevention program (180 min);*			X_s4_	X_s5_								
	***Session 6/7*** *– Plan-Do-Study-Act cycles 1 and 2 (90 min each);*							X_p1_		X_p2_			
	***Session 8*** *– Refresher training (180 min);*											X_s8_	
	***Session 9*** *– Plan-Do-Study-Act cycle 3, plus engagement of physicians in the Sequential group (90 min);*												X_p3_ *M*_*2*_
**YEAR 2 (March 2018–March 2019)**		**MONTH**											
**Implementation strategy component**	**Actions conducted**	13 March	14 April	15 May	16 June	17 July	18 Aug	19 Sptb	20 Octb	21 Nov	22 Dec	23 Jan	24 Feb
***Continuation of …*** **Strengthening of local leadership**	**Ongoing support meetings** (4 h/meeting)	X_Lsm6_		X_Lsm7_				X_Lsm8_		X_Lsm9_		X_Lsm10_	
***Continuation of …*Collaborative planning of the intervention program**	***Session 10*** *– Standardization of the local program (90 min)*	X_s10_■											
***Ongoing sustainability*** *Goals****:*** *to continually support and assess innovation being put into practice Discrete implementation strategies****:*** *Develop quality monitoring systems (ERIC 27); Audit and provide Feedback (ERIC 5);*	***Regular audits and ongoing facilitation sessions*** *(6 sessions, 90 min each)*			X_ms1_ M_4.1_					X_ms2_		X_ms3_ M_4.2_		X_ms4_ M_4.3_
	***Ongoing supportive training***			*M*_*3*_					X *M*_*5*_				
**YEAR 3 (March 2019–May 2019)**		**MONTH**											
**Implementation strategy component**	**Actions conducted**	25 March		26 April		27 May		28 June					
***Continuation of …*Strengthening of local leadership**	**Ongoing support meetings** (4 h/meeting)					X_Lsm11_							
***Continuation of … Ongoing sustainability***	***Regular audits and ongoing facilitation sessions*** *(6 sessions, 90 min each)*					Xms5 *M6*							

*X* Completed, *M* Modification, ■ Type 2 diabetes prevention program initiation, X _Lt_ Leader training, *X*_*Lsm*_ Leader support monitoring sessions_,_ *X*_*s*_ Core implementation session at each center_,_ *X*_*p*_ PDSA cycle session_,_ *X*_*ms*_ Regular audits and ongoing facilitation sessions

**Table 3:** Reporting modifications of the PVS-PREDIAPS implementation strategy

***1. Added actions***			
**Modification/Description**	*Additional training session in the recommended clinical intervention and in the ICT tool (M*_*1*_)	*Additional session for physicians’ involvement in the Sequential Group (M*_*2*_)	*Additional training session in the recommended clinical intervention and in the ICT for new professionals (M*_*3*_)
**When did the modification occur?**	Month 1 of the implementation phase	Month 11 of the implementation phase	Month 20–21 of the implementation phase
**Were modifications planned?**	Unplanned and reactive	Unplanned and reactive	Unplanned and reactive
**Who participated in the decision to modify? Who made the ultimate decision?**	Implementation team, formed by healthcare professionals of the center and the external facilitator	Local leader and the external facilitator	Implementation team, formed by healthcare professionals of the center and the external facilitator
**What was modified?**	The dose of the training component by repeating the former planned action/session	The dose and the actors involved, as the session for “involvement of physicians” (PDSA session #3) was repeated adding the presence of the integrated healthcare organization management staff	An additional training session was to newly incorporated professionals was offered, scheduled and performed
**At what level of delivery (for whom/what is the modification made)?**	Healthcare professionals of one PC of the Sequential group (Sv)	One PC of the Sequential group (Sv)	Few new professionals from 4 centers: 1 from the Global (A, *n* = 3); 3 from the Sequential (Sv, n = 1; Z, *n* = 2; So, n = 1)
**What is the nature of the content modification?**	An additional 90-min session was performed on the recommended clinical health promotion intervention and the ICT healthy lifestyle promotion tool	An additional 90-min session with all PC professionals with the participation of representatives of the management of the integrated healthcare organization	An additional 90-min session was performed on the recommended clinical health promotion intervention and the ICT healthy lifestyle promotion tool
**Relationship fidelity/core elements?**	Fidelity consistent/core elements or functions preserved	Fidelity consistent/core elements or functions preserved	Unknown
**What was the goal?**	Increase healthcare professional’s knowledge and skills to provide the recommended clinical intervention	Maximize number of physicians engaged	Train newly incorporated professionals to provide the recommended clinical intervention
**What are the reasons for the modifications?**	Perceived difficulty of the providing the recommended intervention through the ICT healthy lifestyle promotion tool	Lack of attendance to the planned session	Staff turnover
***2. Actions not or only partially delivered***			
**Modification/Description**	*Failed to organize and conduct the planned ongoing supportive training session (M*_*5*_*)*	*Failed to attend to regular audits and ongoing facilitation sessions (M*_*4*_*)*	*Failed to organize and conduct the planned number of regular audits and ongoing facilitation sessions (M*_*6)*_
**When did the modification occur?**	Month 20 in the post-implementation phase	Months 15, 22 and 24 in the post-implementation phase	Post-implementation phase
**Were adaptations planned?**	Unplanned and reactive	Unplanned and reactive	Unplanned and reactive
**Who participated in the decision to modify? Who made the ultimate decision?**	Local leader and healthcare professionals of the center	Local leader and healthcare professionals of the center	Implementation team, formed by healthcare professionals of the center and the external facilitator
**What was modified?**	Planned training session suspended	Planned and scheduled sessions suspended	Planned sessions suspended
**At what level of delivery (for whom/what is the modification made)?**	2 centers in the Sequential group (Eg, So) and 2 in the Global group (A, Z) failed	2 PC in the Sequential group (Eg, Z) and one in the Global group (I) failed once; 1 PC in the Sequential group (Er) failed twice	All centers
**What is the nature of the content modification?**	No modification required	No modification required	No modification required
**Relationship fidelity/core elements?**	Fidelity inconsistent as this was a core element within the planned strategy	Fidelity inconsistent as this was a core element within the planned strategy	Fidelity inconsistent as this was a core element within the planned strategy
**What was the goal?**	Increase healthcare professionals’ knowledge and skills to provide the recommended clinical intervention	Provide ongoing support and assess progress in the implementation process	Provide ongoing support and assess progress in the implementation process
**What are the reasons for the modifications?**	Difficulty for scheduling meetings due to work overload or other competing priorities	Difficulty for scheduling meetings due to work overload or other competing priorities	Difficulty for scheduling meetings due to work overload or other competing priorities

In the process of executing the strategy, various unplanned and reactive modifications were made (see Table 3). Specifically, three actions were carried out in addition to those planned: two were related to training, one of these being a session for one center and the other for new nursing staff from several centers; and one was a repeat session for encouraging participation of medical staff in one of the Sequential strategy centers, on this occasion involving managerial staff of the integrated healthcare organization, seeking to boost the involvement of physicians. All of these modifications were requested by the centers themselves via the local leader. On the other hand, three of the planned actions were not carried out as intended. Specifically, of the planned six ongoing monitoring and facilitation sessions, a maximum of five were held, with fewer in some centers, mainly due to scheduling constraints and heavy workloads. Additionally, not all centers managed to organize and run one of the planned Ongoing supportive training sessions.

Table 2 displays the original implementation plan involving 11 ERIC discrete implementation strategies and the intervention mapping, which consisted of a combination of 3 ERIC discrete strategies, yielding a total of 14 discrete strategies. In the group surveys/interviews regarding the perceived usefulness of the implementation strategies, the healthcare professionals recognized half of these 14 planned strategies. Appendix Table 5 lists the 14 planned strategies, as well as 3 additional ERIC implementation strategies that were perceived by the healthcare professionals to be part of the implementation process. In general, the strategies that were identified were rated positively, with 10 ERIC strategies described by the healthcare professionals as useful for their own professional development, valuable for the optimization process, and/or specifically helpful to build competence, engage professionals, and/or enhance inter-professional collaboration. Room for improvement was noted for only two ERIC strategies delivered - audit and feedback (ERIC 5) and conducting educational meetings (ERIC 15). While centers assigned to both groups identified the need for increased frequency, length, or quality of educational sessions, only one center (assigned to the Global arm) indicated a need for more audit and feedback sessions. Few differences were observed in the perception of strategies received between centers assigned to each arm. Healthcare providers in the centers assigned to the Global strategy identified the importance of conducting cyclical small tests of change (ERIC 14) and revising professional roles (ERIC 59), while none in the Sequential group identified these strategies. In contrast, one Sequential group center noted the value of promoting adaptability (ERIC Strategy 51), while no Global group centers did. Facilitation (ERIC 33) and need to mandate change (ERIC 44) were identified as important by more Global than Sequential arm centers, while providing audit and feedback (ERIC 5) and conducting local needs assessment (ERIC 18) were identified by more Sequential than Global arm centers. See Appendix Table 5.

## Discussion

Fidelity evaluation is necessary to advance knowledge and understanding about the effectiveness of implementation strategies designed to facilitate adoption of evidence-based interventions and practices [2, 4]. The PREDIAPS project seeks to generate scientific evidence concerning the optimization of healthcare practice in primary prevention of T2D in Osakidetza PC centers through the application of implementation science as a way to achieve feasible, sustainable and effective translation of the recommended evidence-based clinical intervention to clinical practice [15]. A first step in this process is to ascertain whether the implementation strategy and the procedures for putting it into practice in the groups and centers compared has been executed as planned or there have been modification or variations that could have an impact on the outcomes of interest and in the future reproducibility of the study [4, 5, 8]. Considering that, we lack a specific framework to guide implementation strategy-level fidelity evaluations, we have adopted the framework for fidelity evaluations stated by Dane and Dusensbury with the purpose of evaluating and reporting on the fidelity of PVS-PREDIAPS implementation strategy and the two procedures for its deployment [15]. This framework considers the following elements in fidelity evaluations: adherence, dose, quality of delivery, participant responsiveness and program differentiation. Results of the present study seem to indicate that the PVS-PREDIAPS implementation strategy to improve T2D primary prevention has been carried out with high degree of fidelity. Despite some differential exposure to overall strategy within the nursing staff of compared groups, professionals involved have been notably exposed to the implementation strategy and the planned program differentiation related to engagement of professionals and deployment of the implementation strategy has been attained.

Part of the present evaluation of PVS-PREDIAPS implementation strategy’s fidelity involves examining the implementation strategy dosage, that is, the degree of passive exposure to the planned implementation strategies and actions. In general, we can state that the professionals involved in both comparison groups had a notably high degree of exposure to the implementation strategy and that, as planned, the procedures for involving the professional groups and delivering the PVS-PREDIAPS strategy actions were executed differently in the two arms. Additionally, the exposure indicators suggest that professionals assigned to execute the strategy through sequential engagement of colleagues (starting with nurses who later sought the engagement of the physicians) had an unexpectedly higher degree of exposure to the implementation strategy actions, particularly in the case of nurses, the staff responsible for providing the active element of the clinical intervention. As described in the literature, commonly reported obstacles faced by physicians to fully engaging in implementation actions included heavy workload, staff turnover, difficulties in investing time and effort improvement initiatives beyond providing care, and existing practice priorities [21–23].

Dosage can also be evaluated subjectively, in terms of perceived receipt of the implementation strategies by the healthcare professionals. Although all healthcare professionals participating in the PREDIAPS trial were exposed to the 14 ERIC discrete implementation strategies with minor modifications, our qualitative evaluation of their experience indicates that they only recognized having received half of them. Many of the strategies that did not emerge from the surveys and group discussion related to “more structural” implementation actions like developing a formal implementation blueprint, changing record systems, or developing and organizing quality monitoring systems. This may be due to the fact that healthcare professionals are not implementation specialists and did not pay attention to or notice these changes. They also failed to identify several “ongoing” implementation activities, such as ongoing training and ongoing support, and local discussion and consensus sessions (collaborative modeling sessions). It is possible that these activities were seen to be typical, or standard, implementation tools that were too obvious to mention in the evaluation session. It is also possible that these strategies were not strong enough to be detectable. In any case, differential participation or exposure to the strategy could compromise the future implementation of the clinical intervention that we are seeking to promote [24]. Therefore, future analysis and interpretation of the main outcomes of interest should be adjusted for the degree of participation of professionals in general and/or exposure to the actions of the implementation strategy as a function of study arm and professional group [13, 23].

A second factor of interest in the evaluation of the fidelity of the execution of an implementation strategy is the extent of changes and adjustments that may have been made in the process of putting it into practice. Nevertheless, an adequate fit between “fidelity” of the strategy and the necessary “adaptability” to the local context of centers remains a great challenge in implementation trials [25]. Few modifications were made to the PVS-PREDIAPS implementation strategy. With respect to the planned implementation strategy [15], it proved unfeasible to carry out some activities, for example, the total number of planned monitoring and ongoing facilitation sessions. As commented previously, difficulty in scheduling meetings due to work overload or other competing priorities are common barriers faced by facilitators in improvement initiatives [23, 26]. There was demand among professionals for additional actions related to specific core strategies within the overall implementation strategy like training actions regarding the clinical intervention. Contextual factors, for example, site characteristics, needs and priorities are considered to be among the main drivers for tailoring implementation strategies [27], and one of the approaches used is to permit flexibility in order to enhance alignment and involvement while offering support and guidance towards change [26, 27].

Regarding participant responsiveness, all the ERIC discrete implementation strategies identified by the healthcare professionals in the evaluation session were described as beneficial. Specifically, 10 ERIC discrete strategies were perceived to be useful by at least one center. Such a positive evaluation is indicative of the high quality of the delivery of all strategies identified. Notably, however, two of these 10 strategies (conducting educational meetings and audit and feedback) were also mentioned as needing improvement by at least one individual in five of the nine centers. Nonetheless, the same two strategies were the most mentioned overall, and therefore also the most positively evaluated. The high frequency of mention within the evaluation session likely correlates with high exposure to these strategies, and this may imply more time for critical thinking about these specific strategies that were an important part of the implementation plan.

Interestingly, healthcare professionals also perceived receipt of ERIC strategies that were not necessarily specified a priori in the implementation plan or incorporated as part of a planned modification. These previously unidentified strategies included creating a learning collaborative, facilitation, and the mandating of change. Given the emphasis of the implementation strategy on facilitation to create a learning collaborative to develop and adapt the implementation strategy to an individual center’s context, it is not surprising that these two strategies were identified by at least half of the centers. The positive evaluation of these strategies seems to indicate the perceived value of external support and building teamwork in the successful implementation of complex interventions in the healthcare setting [26, 28]. Moreover, the participants felt that mandating change (socially and/or organizationally) fostered engagement in half the centers in the Global arm and built competence in one center in the Sequential arm. In the PREDIAPS trial, the perception of these additional discrete implementation strategies provides further evidence of an appropriate dose having been received and suggests further ecological validity of the overall implementation plan. Lastly, the lack of large qualitative differences in perceived receipt of implementation strategies observed between centers assigned to the Global and Sequential collaborative processes provides subjective evidence that exposure to planned implementation strategies seems to have been fairly similar, regardless of the deployment procedure.

The present study has some important limitations. The first, and possibly most important, is the small number of participating centers, which limits the potential generalizability of the findings to other PC centers in the Basque Country or other health systems. Second, the emphasis on the beneficial aspects of the implementation strategies identified in the qualitative inquiry may have biased our evaluation of participant responsiveness. The qualitative evaluation was carried out exclusively with the local leaders, who, though best positioned to provide related information, might have a different perspective to that of their colleagues. Furthermore, most of the open-ended questions posed in the evaluation session tended to be phrased positively in terms of usefulness. Future studies of participant responsiveness should consider researcher/interviewer bias in the design and realization of the evaluation. In interpreting our results, potential social desirability bias in participant responses should also not be ruled out.

Despite these limitations, a major strength of this study is the nature of the results obtained regarding fidelity, as they demonstrate that professionals involved were capable of identifying and rating the implementation actions conducted. Moreover, the data presented in this manuscript provides a practical example from the PREDIAPS trial that brings to life the core components of fidelity assembled from various existing frameworks. These components include adherence to the planned implementation strategies, dose/exposure to the strategies, quality of delivery, participant responsiveness to the strategies received, modifications made and program differentiation. Given the lack of operational definitions and existing frameworks to evaluate the fidelity of implementation strategies, this paper helps advance scientific research on fidelity.

The present fidelity evaluation has fulfilled some of its most important goals. In this sense, it seems to confirm the high quality of the implementation of the PVS-PREDIAPS strategy and of the two procedures for its deployment. Further, it will help to explain and interpret future results of the PREDIAPS trial, by rejecting the possibility of an implementation failure and by informing about potential confounding factors due to differences observed between comparison groups, these being potentially associated with both exposure and results. Lastly, it points out to some core elements of the implementation strategy that should be improved for future dissemination and scale-up, as for example the training component. In this sense, there is missed opportunity regarding the assessment of the training component of the strategy (eg., overall quality, satisfaction, etc.) that could lead to an improvement to ensure that professionals of any background and skills to be appropriately trained to deliver the intervention in a standardized way. Nevertheless, the majority of the demanded modifications were related to additional actions related to training in the clinical intervention.

## Conclusions

The PVS-PREDIAPS implementation strategy to improve T2D primary prevention of the collaborating PC centers has been carried out with high degree of fidelity in each of the main measured dimensions. Despite some differential exposure to overall strategy in comparison groups, mainly in the nursing staff, professionals involved in both comparison groups have been notably exposed to the implementation strategy and the planned program differentiation related to engagement of professionals and deployment of the implementation strategy has been attained. Some minor unplanned reactive modifications have been required within the strategy responding to contextual circumstances related to centers’ work overload. Future analysis and interpretation of results pertaining to the main study will need to consider the mentioned differences in actual degree of exposure to implementation strategy’s actions within group and professional levels. The framework for fidelity evaluations stated by Dane [8] and adapted by Dusenbury [5], is a valuable framework that can be used to evaluate the implementation strategy-level fidelity. The FRAME framework [18] may be a useful complement in order to identify and report modifications made within the planned implementation strategy.

## Figures and Tables

**Fig. 1.**
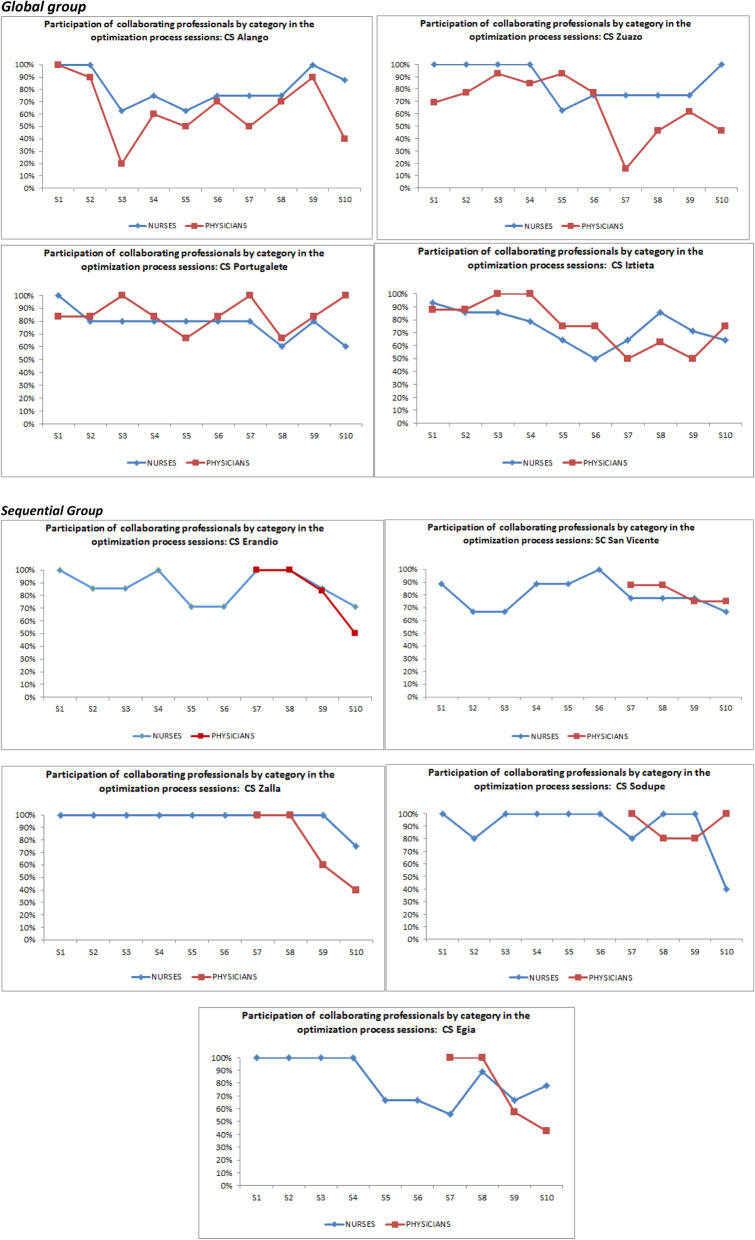
Participation of PC professionals in the PREDIAPS strategy in the planned implementation sessions (*n* = 10)

## Data Availability

Since data supporting the present study will mostly concern to process data and reports from specific professionals of the Basque Health Service-Osakidetza, it will be only shared upon justified request to the study guarantors.

## Appendix

**Table 4:** The PVS-PREDIAPS clinical intervention reported according to the Template for Intervention Description and Replication (TIDieR) checklist

Brief name	PVS-PREDIAPS (from the Spanish “Prescribe Vida Saludable-Prevención diabetes en Atención Primaria de Salud”)
Rationale	The 5 A’s (Ask, Advise, Agree, Assist, and Arrange follow-up) intervention framework was used to standardize the provision of the evidence-based behavior modification techniques used to promote changes in physical activity (150 min of moderate physical activity a week) and diet (Mediterranean-type healthy diet) to prevent type-2 diabetes in high-risk patients
Materials and Procedures	Intervention delivery procedure The multiple active intervention components and strategies are structured following the 5 A’s (Ask, Advise, Agree, Assist, and Arrange follow-up) intervention framework *Assess* Assess the risk of type-2 diabetes to identify patients eligible for the intervention and compliance with recommended levels of physical activity and daily servings of fruit and vegetables using the PVS screening questionnaire. *Advise* Provide clear, specific and personalized advice on changing lifestyles, including information on health risks and benefits *Agree* Select in collaboration with the patient the lifestyles change objectives, based on the preferences, interests and capacity for the change of the patient *Assist* Cooperatively design an action plan with the patient that determines the specific objectives of lifestyle change, including the identification of possible barriers, problem solving, and coping strategies to facilitate behavior change. The lifestyle change plan designed is provided to the patient in the form of a printed prescription. *Arrange follow-up* Organize follow-up (in person or by phone) every 3 months up to one year to provide ongoing assistance and support, and to adjust the action plan as necessary. Provided supporting materials Patients at high risk of type-2 diabetes received printed materials to support health care professionals’ promotion intervention in order to foster patients’ motivation to change lifestyles and to provide guidance in how to perform lifestyle change successfully. Specifically, those receiving healthy diet advice or prescription (see the intervention procedure bellow) received a printed version of the Spanish Society of Community Nutrition (SENC from the Spanish “*Sociedad Española de Nutrición Comunitaria*”) document [web Access: https://www.nutricioncomunitaria.org/es/otras-publicaciones]. Those receiving the physical activity promotion intervention were provided with a *PVS-Physical Activity* pamphlet with information about benefits of physical activity and risk of inactivity, and a summary of potential barriers and coping strategies to overcome them.
Who provided the intervention?	In general, family physicians performed the screening and referred high-risk patients identified to nurses for delivery of the healthy lifestyle promotion. Nurses first asked patients about their lifestyle and then provided personalized advice tailored to the patient’s needs, encouraging individuals motivated to make lifestyle changes to attend an additional consultation at which a lifestyle change is prescribed and a personalized plan for modifying habits and monitoring change achieved over time is developed in collaboration with the patient. Though the distribution of the components of the intervention is established at the level of PC team, physicians were allowed to opt for a different approach (e.g., also assessing lifestyle behaviors and providing advice, in addition to screening for T2D risk).
How? Where? When?	The healthy lifestyle promotion intervention was delivered in routine context of primary care during opportunistic or programmed visits.
Tailoring	Although the intervention is based on a shared decision-making process in relation to behavior change, it takes into account the patient’s willingness to change and their autonomy in a context of cordiality. Thus, those who are not committed to the possibility of making a change in behavior after receiving the advice and being confronted with the possibility of making an additional consultation to design a personalized plan to change habits, receive the support material and are summoned to address the issue on a future visit. Those committed to change who accept the additional appointment for the design of a personalized plan for change habits are those who receive the intervention fully, including the follow-up.
Modifications	No modifications were performed during the trial
How well the intervention was delivered	An information and communication tool integrated in the electronic health record was developed in order to help and guide healthcare professionals to deliver the healthy lifestyles promotion intervention in a standardized way. The tool includes the following functions: - Facilitates the assessment of lifestyle behaviors, tracks the clinical diagnosis of compliance with current recommendations, and enhances motivation for changing behaviors. - Helps to identify sub-populations at high risk of developing chronic diseases, based on data stored in clinical databases. - Guides professionals in the provision of personalized medical advice adapted to the patient and offers an outline for the prescription of personalized plans to modify lifestyle behaviors. - Registers and stores data of the actions carried out in each person’s EHR to promote follow-up.

**Table 5:** Evaluation of PVS-PREDIAPS discrete implementation strategies as cataloged by the Expert Recommendations for Implementing Change (ERIC) taxonomy

Strategies in *italics* were those identified a priori to be used as part of implementation plan (X) Implementation strategy identified as useful for own professional development (+) Implementation strategy considered valuable for the optimization process (B) Implementation strategy needed to foster competence building (E) Implementation strategy needed to facilitate engagement of professionals (C) Implementation strategy needed for inter-professional collaboration (−) Implementation strategy identified as needing improvement									
	Global group				Sequential group				
PREDIAPS-ERIC Strategies	PC1 A	PC2 Zu	PC3 Iz	PC4 P	PC5 Er	PC6 Sv	PC7 Eg	PC Za	PC So
Pre-planned ERIC Strategies									
5. Audit and provide feedback	+		X -		B	X +		X	+
12. Change record systems									
14. Conduct cyclical small tests of change	X +	+							
15. Conduct educational meetings	X +	X -	X +	X -	X	X ++ −	X +	X	X - B
17. Conduct local consensus discussions									
18. Conduct local needs assessment			X					X	X
19. Conduct on-going training									
23. Develop a formal implementation blueprint									
27. Develop and organize quality monitoring systems									
48. Organize clinician implementation team meetings									
51. Promote adaptability								+	
55. On-going support for implementation									
57. Recruit, designate, train for leadership	E							C	
59. Revise professional roles	C								
Additional ERIC Strategies Perceived									
20. Create a learning collaborative		X	+	XX +	X		+	X	X +
33. Facilitation	X	+		+		X +			
44. Mandate change	E	E			B				

## Abbreviations

T2D: Type-2 diabetes
PC: Primary Care
MRC: Medical Research Council
FRAME: Framework for Reporting Adaptations and Modifications-Enhanced
PDSA: Plan-Do-Study-Act
ERIC: Expert Recommendations for Implementing Change
